# SIV infection of rhesus macaques of Chinese origin: a suitable model for HIV infection in humans

**DOI:** 10.1186/1742-4690-10-89

**Published:** 2013-08-15

**Authors:** Yu Zhou, Rong Bao, Nancy L Haigwood, Yuri Persidsky, Wen-zhe Ho

**Affiliations:** 1 The Center for Animal Experiment/ ABSL-III Laboratory, State Key Laboratory of Virology, Wuhan University School of Medicine, Wuhan, Hubei 430071, P.R. China; 2 Oregon National Primate Research Center, Oregon Health & Science University, Beaverton, OR 97006, USA; 3 Department of Pathology and Laboratory Medicine, Temple University School of Medicine, Philadelphia, PA 19140, USA

**Keywords:** Simian immunodeficiency virus (SIV), Nonhuman primate (NHP), Chinese rhesus macaques (RM), Indian RM, HIV/AIDS

## Abstract

Simian immunodeficiency virus (SIV) infection of Indian-origin rhesus macaques (RM) has been widely used as a well-established nonhuman primate (NHP) model for HIV/AIDS research. However, there have been a growing number of studies using Chinese RM to evaluate immunopathogenesis of SIV infection. In this paper, we have for the first time reviewed and discussed the major publications related to SIV or SHIV infection of Chinese RM in the past decades. We have compared the differences in the pathogenesis of SIV infection between Chinese RM and Indian RM with regard to viral infection, immunological response, and host genetic background. Given AIDS is a disease that affects humans of diverse origins, it is of importance to study animals with different geographical background. Therefore, to examine and compare results obtained from RM models of Indian and Chinese origins should lead to further validation and improvement of these animal models for HIV/AIDS research.

## Review

### Introduction

Nonhuman primate (NHP) models have been widely used to study the pathogenesis, vaccine and therapeutic intervention of HIV/AIDS since the isolation of HIV and simian immunodeficiency virus (SIV) strains [1-3]. The first use of SIV infection of rhesus macaques (RM) for testing a candidate HIV vaccine was reported in 1989 [4], after which there has been a dramatic increase in the utilization of rhesus and other macaque models in the HIV field. Multiple factors contribute to the high demand for macaques. First, there are still a multitude of preclinical HIV drugs and vaccine approaches and modalities being tested, which requires a large number of animals [5]. Second, it has been recognized that repeated mucosal exposure to low dose of SIV in rhesus macaques resembles HIV infection in humans [6]. Once the set-point phase is reached following mucosal SIV challenge, the level of viral load predicts the rate of progression to AIDS [7-10]. As a result, attempts to detect subtle differences in virological and immunological outcomes in vaccinated animals challenged by low dose mucosal routes of infection have greatly increased the number of animals required for each vaccine study [11]. Third, NHP models, particularly RM, have been widely used to investigate the events of SIV infection with respect to viral dynamics, immune responses, and changes in the pool of CD4^+^ cells, which further enhances the need for suitable macaque hosts for studies of HIV pathogenesis [12].

There are three available macaque species [rhesus (*Macaca mulatta*), cynomolgus (*Macaca fascicularis*), and pigtail (*Macaca nemestrina*)] that are susceptible to SIV infection and develop AIDS-like disease [1]. Rhesus macaques of Indian origin have often been used for SIV infection as they are the primary species provided by the breeding facilities in the USA [1]. SIV infection in Indian-origin RM has become the most established model of HIV infection and AIDS-related research due to development of tools to study immune responses in depth [13] and the concomitant wealth of experimental data available. Additional research has utilized the chimeric virus, SHIV, for experiments that require HIV proteins, such as HIV envelope-based SHIVs for testing of human neutralizing antibodies on Indian RM, summarized in a recent review [14]. As a mainstay of nonhuman primate models in HIV/SIV research, the availability of Indian RM model has contributed greatly to our understanding of HIV transmission, pathogenesis, prevention and therapy. However, like any existing animal models for studying human diseases, the Indian RM model has its limitations. The biological discrepancies between human and simian AIDS reveal certain drawbacks of Indian RM in HIV research. For example, the progress to disease is more aggressive in Indian RM when infected with most if not all SIV strains, and the plasma viral loads are higher than HIV infection of humans [15-17]. Also, with regard to the dynamics of the CD4^+^CCR5^+^ memory T cells, an important marker for HIV/SIV disease progression [12,18], there are some differences between SIV infection in Indian macaques and HIV infection in humans.

Belonging to the same species as Indian RM, Chinese RM have also been used in HIV/SIV research. Over the past decades, an increasing body of evidence shows that similar to RM of Indian origin, Chinese RM can be readily infected by SIV through different inoculation routes, and were proved to be useful in evaluating pathogenesis, vaccine, and therapeutic strategies for HIV/AIDS studies [15-18]. However, because of different course of SIV infection in Chinese RM compared with that in Indian RM, the use of Chinese RM in AIDS research has been limited. In this review, we describe and discuss the major publications related to SIV infection of Chinese RM over the past decades and compare the differences in SIV and SHIV infection between Chinese RM and its Indian-origin counterpart.

### Experimental evidence for SIV/SHIV infection of Chinese RM

Early experimental evidence for successful infection of Chinese RM by SIV came from a study by Joag et al. [19], in which all six Chinese RM enrolled in the study developed acute plasma viremia after SIVmac239 inoculation [19]. Subsequent research demonstrated that the course of SIV infection in Chinese RM is somewhat different from that seen in Indian RM. Ling et al. utilized SIVmac239 to intravenously infect ten Chinese RM and found that all Chinese RM quickly developed viremia with magnitudes and kinetics of viral load similar to HIV infection of humans [20]. Infection of Chinese RM by SIVmac239 was further demonstrated by several other groups (Table 1). In addition to SIVmac239, infection of Chinese RM by SIVmac251, SHIV and primary virulent isolate SIV/ DeltaB670 [21], has been documented (Tables 1 and 2). Marthas and colleagues [22] found that there was no significant difference between Indian RM and Chinese RM with regard to the number of animals that were infected with intravaginal inoculation of SIV251. They observed that, although there was considerable overlap in the range of viral loads between both Indian and Chinese RM, the variation in viral loads among Indian RM was greater than that among Chinese RM [22]. In addition, SIV-infected Chinese RM tended to have the lower end of the range of viral loads compared with infected Indian RM [22]. Chen et al. compared infection of Chinese RM with two different SIV strains, SIVmac251 and SIVmac239, and observed that SIVmac239 infection was more pathogenic, as it caused more aggressive disease than SIVmac251 [9], suggesting the potential variability of bio-clinical parameters in Chinese RM when infected by different strains of SIV. Wang et al. [23] showed that a CCR5-tropic chimeric SHIV (SHIV_B’WHU_) could replicate in Chinese RM peripheral blood and cause acute infection of these animals with no significant changes in viral tropism and sequences after infection. Miyake et al. [24] infected Chinese RM with pathogenic SHIV (SHIV-C2/1-KS661c) by intrarectal inoculation and observed rapid dissemination of SIV to multiple organs, including rectum, thymus and axillary lymph node (LN) as early as three days post infection. Further analysis of viral kinetics showed that infectious virus was first detected at day six post infection (p.i.) and high levels of proviral DNA and infectious virus were both detected by 13 days p.i.. However, by 27 days p.i., SIV loads decreased dramatically. Pal et al. [25] demonstrated that replication competent SHIV encoded HIV reverse transcriptase gene (RT SHIV) could be transmitted efficiently via the vaginal route in Chinese RM. Some of these infected animals had persisting viremia for one year. In a co-infection model, Chenine et al. showed that Chinese RM were able to be infected by clade C SHIV (SHIV-C) and the infection was significantly facilitated by the parasite co-infection [26].

**Table 1:** Clinical parameters in SIV-infected Chinese RM

**Animal no.**	Age (Year)^*^	**Gender**	Inoculation^**^	**SIV strains**	**Dose AID**_**50**_**/TCID**_50_^***^	**Peak viral load**	Survival time^****^	**Refs**
10	3–10	7F/3M	I.V.	mac239	10^2^ TCID_50_	3.5 × 10^6^–6.0 × 10^7^	>34W	[20]
50	3–6	78M/72F	I.R.	mac239	5 × 10^5^ TCID_50_	10^7^	>99W (25/50)	[9]
50			I.V.	mac239	2 × 10^2^ TCID_50_	10^7^	>117W (25/50)	
8	5–11	M	I.V.	mac239	5 × 10^3^ TCID_50_	10^6^–10^8^	>69W (4/8)	[27]
4	Adult	M	I.V.	mac239	5 × 10^3^ TCID_50_	5 × 10^6^–1 × 10^8^	NP	[28]
3	3–6	2M/1F	I.V.	mac239	10^3^ TCID_50_	3.2 × 10^6^–3.2 × 10^7^	>25W	[29]
3	3–8	Mixed	I.R.	mac239	2.5 × 10^3^ TCID_50_	10^6^–10^7^	>44W	[30]
9	NP	NP	I.V.	mac239	10^2^ TCID_50_	~10^6^–10^8^	104–354W	[31]
7				mac239	2 × 10^6^ copies		31–172W	
1	Adult	F	IVAG × 3	mac239	~10^5^ TCID_50_	~7.9 × 10^6^	>46W	[32]
4	NP	NP	I.V.	mac251	5 × 10^2^ TCID_50_	10^6^–2 × 10^8^	>18M	[33]
8				mac239	NP		>36M	
24	5	M	I.R.	mac239	10^3^ TCID_50_	10^5^–5 × 10^7^	>24W	[34]
2	5–6	F	IVAG	mac239	10^5^ TCID_50_	2.5 × 10^6^–3.2 × 10^6^	>25W	[35]
50	3–6	NP	I.V.	mac251	2 × 10^2^ TCID_50_	5 × 10^6^	>118W (27/50)	[9]
14	4–5	F	I.V.	mac251	10^5^ TCID_50_	1.1 × 10^5^–1.2 × 10^8^	NP	[22]
10	4–6	F	I.V.	mac251	50 TCID_50_	2.4 × 10^4^–1.4 × 10^6^	51-72W	[36]
6	NP	NP	I.V.	mac251	10 AID_50_	7.0 × 10^5^–3.2 × 10^7^	NP	[37]
10	7.5–9.5	NP	I.V.	mac251	10 AID_50_	2.6 × 10^5^–3.6 × 10^7^	NP	[12]
12	NP	NP	I.V.	mac251	10 AID_50_	10^5^–10^8^	5–108W (killed)	[38]
4	2–4	mixed	I.V.	mac251	5 AID_100_	2.7 × 10^7^–7.6 × 10^7^	>42W	[39]
8	NP	NP	I.V	mac251	10 AID_50_	6.7 × 10^5^–3.2 × 10^7^	>154W (4/8)	[40]

^*^NP: The information is not provided in the paper;

^**^I.R.: intrarectally; I.V.: intravenously; IVAG: intravaginally;

^***^TCID_50_: 50% tissue culture infective dose; AID_50_ : 50% animal infectious doses; Viral titer was quantified in different cells;

^****^W: Weeks; M: Months.

**Table 2:** Clinical parameters in SHIV-infected Chinese RM

**Animal no.**	Age (Year)^*^	**Gender**	Inoculation^**^	**SHIV strains**	**Dose AID**_**50**_**/TCID**_50_^***^	**Peak viral load in plasma**	Survival time (weeks)^****^	**Ref**
2	3.5–4.5	mixed	I.R.	89.6P	40 AID_50_	8 × 10^5^–7 × 10^6^	>24W	[41]
10	6.1–7.1	NP	I.V.	89.6P	10^3^AID_50_	1 × 10^7^	>36W	[42]
6	4–5	NP	I.V.	89.6P	20 AID_50_	~2 × 10^5^–3 × 10^6^	>40W (5/6)	[43]
6	4–8	NP	I.V.	89.6P	10^3^ AID_50_	NP	NP	[44]
6	NP	F	IVAG (13 times)	SF162P3	20, 30 TCID_50_	6.3 × 10^5^–1.6 × 10^7^	>29W	[45]
8	3–5	F	I.V.	SF162P4	18 AID_50_	1.6 × 10^5^–2.5 × 10^6^	>10W	[46]
8	5–6	F	I.V.	SF162P4	22 TCID_50_	1.6 × 10^5^–3.2 × 10^6^	>10W	[47]
12	3.5–7	F	IVAG (6 times)	SF162P3	3 × 10^2^ TCID_50_	1.1 × 10^6^–3.5 × 10^6^	N/P	[48]
4	Adult	M	I.R.	SF162P3	10 TCID_50_	~10^6^–10^7^	>32W	[49]
10	5–15	F	IVAG	SF162P3	3 × 10^2^ TCID_50_	3.2 × 10^5^–1 × 10^8^	≥10W	[50]
8	NP	7M/1F	I.R. (M) IVAG (F)	SF162p3	10 TCID_50_	NP	NP	[51]
4	8.09 (mean)	NP	I.R.	RT	10^3^ TCID_50_	1 × 10^4^–2 × 10^6^	>20W	[52]
2					10^4^ TCID_50_	5 × 10^5^–2 × 10^7^	>16W	
5	Adult	F	IVAG	RT	10^3^ TCID_50_	2 × 10^6^–2 × 10^7^	>20W	[53]
2					2 × 10^2^ TCID_50_	1 × 10^6^ (1/2)	>20W	
16	4–12	F	IVAG	RT	10^3^ TCID_50_	10^5^–10^7^ (9/16)	>16W	[54]
30						~4 × 10^4^–6 × 10^7^ (22/30)		

^*^NP: The information is not provided in the paper;

^**^I.R.: intrarectally; I.V.: intravenously; IVAG: intravaginally;

^***^TCID_50_: 50% tissue culture infective dose; AID_50_ : 50% animal infectious doses; Viral titer was quantified in different cells;

^****^W: Weeks; M: Months.

In addition to the successful establishment of SIV infection in Chinese RM as described above, investigators have also utilized SIV infection of Chinese RM to recapitulate the host immune activation of HIV infection, including immune cell proliferation, activation, and apoptosis. Several studies have found occurrences of marked immune activation and lymphocyte apoptosis in Chinese RM following SIV infection, which are the key pathogenic factors during SIV infection of RM [10,40,55,56]. A study by Monceaux et al. documented that extensive apoptosis in lymphoid organs indicates rapid AIDS progression during primary SIVmac251 infection in Chinese RM [10]. Cumont et al. [40] also showed that there was an increase in lymphocyte apoptosis in LNs during primary SIVmac251 infection of Chinese RM, the levels of which were associated with the degree of viral replication and the rate of AIDS progression. They further demonstrated that the level of lymphocyte apoptosis in RM of Chinese origin was lower than in those of Indian RM. This study was supported by Viollet et al. [55] who reported that death of CD4^+^ T cells from lymph nodes during primary SIVmac251 infection indicates the rate of disease progression to AIDS in Chinese RM. In study of the CD4^+^ and CD8^+^ T cell dynamics during the asymptomatic phase of SIVmac251 infection of Chinese RM, Monceaux et al. revealed that during the asymptomatic phase, the CD4^+^ T cells were sustained in the axillary LNs, while progressively depleted in the peripheral blood [57]. They also found that during primary SIV infection, the intense CD8^+^ T cell activation and an elevation of optimal effector CD8^+^ T cell function correlate with a poor prognosis for AIDS progression [56]. Similarly, Ho et al. utilized the nef-deleted SIVmac251 to infect the Chinese RM and demonstrated that the attenuated SIVmac251 induced pathological CD4^+^ T cell depletion in at least half of the animals, which was associated with a thymic defect [58]. Our recent studies also revealed that LPS administration induced CD4^+^ T cell activation during SIVmac239 infection of Chinese RM (unpublished data). Apart from lymphocytes, other immune cells such as neutrophils, monocytes and dendritic cells, are also prone to apoptosis in pathogenic SIV infection of Chinese RM [59,60]. Polymorphonuclear neutrophils (PMN) in chronically HIV-infected patients were revealed to be more prone to die [60]. Similarly, neutrophils were also depleted during primary SIV infection in Chinese RM, leading to an early and sustained neutropenia in these animals [37]. Therefore, it was proposed that increased PMN death can be used as a marker to predict destruction of host defense against HIV/SIV infection [37,59].

Collectively, there have been more than 70 peer-reviewed publications to date that clearly demonstrate that Chinese RM are susceptible to infection by various non-adapted SIV strains (mac182, mac251, mac239, delta B670, nef-deleted mac251) as well as by engineered SHIV (89.6, 89.6P, SF162P3, SF162P4, RT). Major clinical parameters for SIV or SHIV infection of Chinese RM in these publications are summarized in Tables 1 and 2, respectively.

### Differences in SIV infection between Chinese RM and Indian RM

#### Overview

Although Chinese RM and Indian RM belong to the same species, they demonstrate differences in physiological and immunologic responses [61-63], as well as genetic background [63-66]. Therefore, it is not surprising to observe differences in infection-induced clinical and immunologic parameters and disease progression rates between Chinese RM and Indian RM following SIV infection. Table 3 summarizes the published observations on the differences in SIV infection between Chinese RM and Indian RM.

**Table 3:** Comparison of key parameters in Chinese RM and Indian RM following SIV infection

**Innoculation route**	**SIV strains (Titer)**	**Rhesus macaque**	**Peak viral load (lg10)**	Set point viral load (lg10)*	**CD4 + T cells (%)**	Antibody titer**	Survival time***	**Ref**
I.V.	mac239	Chinese RM (n = 10)	6.5–7.8	~2.8–6.7	>50% (7 out of 10)	10 out of 10 positive	NP	[20]
	(10^2^ TCID_50_)	Indian RM (n = 4)	7–8	~6–7	<50% (2 out of 4)	1 out of 4	>17W (2/4)	
	mac251	Chinese RM (n = 8)	5.8–7.5	2.5–5.8	NP	NP	>154W (4/8)	[40]
	(10 TCID_50_)	Indian RM (n = 6)	6.9–7.8	4.5–7.3	NP	NP	<52W	
	mac251	Chinese RM (n = 8)	6.47–7.88	3.62–5.70	51%	~10^3^	>43W (8/8)	[8]
	(10^2^ TCID_50_)	Indian RM (n = 15)	6.64–8.30	4.37–7.30	49%	~10^3^	>43W(10/15)	
IVAG	mac251	Chinese RM (n = 10)	7.2	5.0	NP	1.6 × 10^4^–8 × 10^5^	NP	[22]
	(10^5^ TCID_50_)	Indian RM (n = 16)	7.7	6.3	NP	2 × 10^2^–8 × 10^5^	NP	

^*^: Measured at day 35–77;

^**^: Measured at wk15-16;

^****^W: Weeks.

#### Differences in virological impact

Ling et al. demonstrated that although there was little difference in peak viral load between Chinese RM and Indian RM, the former had a significantly lower set point viral load than the latter (~2.8-6.7 vs. 6–7 at lg10 scale) [20]. Such a difference in viral load between Chinese and Indian-origin RM was also observed for SIVmac251 infection at various inoculation doses [8,22,40]. Using SIVmac182, the steady-state plasma viral loads achieved in Indian monkeys were significantly higher than those found in Chinese macaques [67]. A difference in survival between the two origins of RM after SIV infection was also observed. For example, four out of eight Chinese RM survived for longer than 154 weeks after viral infection, whereas in the same study all Indian RM died 54 weeks after infection [40]. There has been strong support in the HIV vaccine field for using Indian RM infected with SIVmac239 due to the high, persistent and reproducible plasma viremia. This property of Indian RM made them the model of preference for vaccines that blunt viremia but do not prevent infection, such as the rhesus cytomegalovirus (CMV) vaccines [68,69]. In these types of challenge studies, very large numbers of RM are needed to determine differences between groups since all animals become infected. A concern over SHIV models has arisen due to their lack of predictability for human trials [70]. However, in examining of HIV vaccines, or testing of passively transferred neutralizing antibodies that can block HIV/SIV infection or enhance immunity [71], the relatively low SHIV load in set point viremia observed in individual Chinese RM is not a disadvantage because significantly different outcomes can be measured with smaller numbers of macaques [72,73].

#### Differences in immunologic response

Chinese RM infected with SIVmac239 demonstrated strong antibody responses; ten out of ten infected Chinese RM had positive antibody responses [20]. In contrast, only one out of four infected Indian RM showed the production of antibodies [20]. A similar pattern was observed for CD4 perseverance, as seven out of ten Chinese RM had >50% CD4/CD8 ratio, while two out of four Indian RM had <50% CD4/CD8 ratio [20]. The study by Monceaux et al. [12] showed that the dynamics of CCR5-expressing CD4+ T cells in the acute phase of SIV-infection in Chinese RM are very similar to those in HIV-infected humans, which is characterized as a transient increase in the proportion of CD4 + CCR5+ T cells in the peripheral blood. In contrast, in SIV-infected Indian RM, the number of CD4 + CCR5+ T cells declined in a more immediate and sustained fashion. The authors further demonstrated that such relative expansion of the CD4+ CCR5+ T cells in SIVmac251-infected Chinese RM was associated with most markers of active disease progression, including high virus replication, overall loss of CD4+ T cell function, time to development of AIDS, and overall survival rate [12]. In addition, Cumont et al. [40] found that Indian RM demonstrated a profound decline in the percentage of CD4^+^ DR^+^ T cells during SIVmac251 infection, while only a transient decrease of CD4^+^DR^+^ T cells was observed in Chinese RM at the peak of virus replication. Moreover, they found that in SIVmac251-infected RM, the frequency of TiA-1-expressing T cells expanded earlier in Indian RM than those in Chinese RM. However, at day 60 p.i., the level of TiA-1 reactivity in Chinese RM became greater than that in Indian RM infected with SIVmac251 [40].

The gastrointestinal (GI) tract is a major site of HIV replication. It has been shown that both acute HIV and SIV infections could result in a dramatic and selective loss of memory CD4^+^ T cells predominantly from the mucosal surfaces in GI tract [74,75]. The drastic but transient depletion of CD4^+^ T cells in the intestine was also found in SIV infection of Chinese RM [76]. Th17 cells are a subset of recently identified CD4^+^ T helper cells that are critical for mucosal immunity. A loss of Th17 cells in the GI tracts has been shown to be associated with disease progression in pathogenic HIV-infected humans, and SIV-infected RM [77-79]. However, in Chinese RM such low frequency of Th17 CD4 cells could be compensated by the emergence of NK T cells that express IL-17 early after infection [76]. In addition to the T cell decline, neutrophils were also found to be depleted early in pathogenic SIV infection in both Indian and Chinese RM [37]. This SIV-infection mediated neutropenia coincided with the peak of viral replication. To compare with Chinese RM, the neutropenia was more severe and more sustained in Indian RM, which is consistent with the overall higher pathogenicity of SIV infection in Indian RM than that in Chinese RM [37].

With respect to the innate antiviral cytokine response to SIV infection, it was found that both pathogenic SIVmac251 and non-pathogenic *nef*-deleted SIVmac251 infection induced more remarkable type I IFN expression in the lymphoid tissues of Indian RM than Chinese RM [80]. The authors also showed that pathogenic SIVmac251 infection induced a stronger type I IFN response in lymphoid tissues than *nef*-deleted SIVmac251, with more aggressive disease progression. They speculated that the increased type I IFN response in pathogenic SIV infection might be due to the increased recruitment of plasmacytoid dendritic cells (pDC) in the peripheral LNs and elevated inflammatory environment. On the other hand, decreased type I IFN expression correlates with reduced pDC recruitment in non-pathogenic SIV infection [80]. In SIV infection of natural hosts, such as African green monkeys (AGMs), the absence of sustained IFN production is related to the absence of viral persistence in LNs at the chronic phase [80].

### The mechanisms for the differences between SIV infected Indian RM and Chinese RM models

The mechanisms for the differences in SIV-induced disease pathogenesis between Chinese RM and Indian RM remain largely unclear. It is likely that multiple factors including viral fitness, immunological responses, and genetic background could all contribute to the divergence between Indian RM and Chinese RM as an NHP model for HIV/AIDS research.

#### Viral fitness

The natural adaption of the SIV strains from Indian to Chinese RM is believed to be critical in determining viral infectivity. It has been shown that compared to Indian RM, SIVmac strains are less well adapted in Chinese RM with limited replication capacity [8,20,22], leading to slower depletion of memory CCR5^+^CD4^+^ T cells in the intestinal mucosa in Chinese RM compared to Indian RM [81]. This assumption is further supported by the observation that in vivo serial passage of SIV in Chinese RM led to enhanced viral infectivity [67]. In addition, increased viral fitness from Indian RM cells may explain the higher replicative ability and faster rate of disease progression in these animals, as some of the earlier studies were performed using virus preparations acquired from repeated serial passage in cells from Indian RM. However, Cumont et al. used an SIVmac251 strain that had been in vitro passaged in peripheral blood mononuclear cells derived from Chinese RM, but still observed an accelerated course of disease in Indian RM, with higher apoptosis in Indian RM than in Chinese RM. This finding suggests that rather than intrinsic properties of SIV, host factors are pivotal determinants of the divergent outcomes of SIV infection in rhesus macaques [40].

#### Host immunological factors

Quantification of the copy numbers of CCL3L, a CCR5 ligand that blocks viral entry, identified that Indian RM usually harbor much lower copy number of CCL3L compared to Chinese RM, suggesting that slower progression of SIV infection in Chinese RM may be a result of a block in entry by increased CCL3L in these animals [82]. In terms of adaptive immune response, it was shown that Chinese RM demonstrated stronger and longer lasting antiviral antibody response as well as SIV-specific CD8 CTL response [81] than Indian RM after SIV infection [20]. To explain the differences between Indian and Chinese RM with respect to how SIV infection affects the dynamics of CD4 + CCR5+ T cells, Monceaux et al. proposed that since the drastic decline of the pool of CD4 + CCR5+ T cells in Indian RM is a key factor for the rapid disease progression during both acute and chronic infection, the direct effect of virus replication [12] might be the major determinant of the immune dysfunction that implicates the disease progression to AIDS and death. In contrast, SIV-infected Chinese RM undergo a relative expansion of the CD4 + CCR5+ T cell pool, the degree of which is correlated with all markers of disease progression. Thus, the effect of immune activation, instead of infection-mediated cell lysis [12], may be a pivotal determinant in the immunological changes related to AIDS progression in Chinese RM.

#### Genetic divergence

In addition to viral and host immunological factors, host genetic factors should also be taken into consideration in understanding of biological differences in SIV infection between the Indian and Chinese RM. Despite belonging to the same species, Chinese RM and Indian RM have diverged into two separate subtypes with marked genetic difference, which could greatly impact viral fitness and host immune responses to the viruses that in turn markedly regulate disease progression in monkeys [9]. The genomic sequencing of Chinese RM and Indian RM has identified numerous distinguished subtype-specific single nucleotide polymorphisms (SNPs) between these monkeys [83]. Further comparisons of Indian RM and Chinese RM mitochondrial DNA (mtDNA) sequences have shown 90% mtDNA genetic heterogeneity [84]. Furthermore, considerable differences in MHC class I and II profiles between Indian RM and Chinese RM greatly shape their adaptive immune responses to viruses and subsequently affect disease pathogenesis. Specific MHC profiles of Chinese RM have been identified to be associated with SIV inhibition [85-88]. The potential differences in MHC profile [63-66] may invoke differences in cellular restriction factors and T cell immune responses. Wambua et al. sequenced a cohort of 12 SIV-infected Chinese RM for the expression of MHC class I alleles and identified that one set of alleles, Mamu-B*1001, and -B*8701, appeared only in some elite controllers and not in normal progressors, whereas several alleles, like Mamu-B*3901, only appeared in animals that progressed to AIDS but not in elite controllers [89]. A recent study [90] suggests that Chinese RM are valuable as representative models of HLA gene diversity and function, and therefore an attractive alternative model for investigating human immune response. However, future studies on direct comparison of MHC profiles between Chinese RM and Indian RM are necessary in order to identify the MHC alleles that are associated with lentivirus-induced disease outcome in these two subspecies of RM.

### Chinese RM as a model for testing new HIV vaccine approaches and therapy

Several studies [41,43,49] have utilized Chinese RM to evaluate novel HIV vaccine approaches and therapies. Stolte-Leeb et al. investigated the protective efficacy of the multigenic DNA prime/MVA boost vaccine approach against mucosal SHIV89.6P infection in Chinese RM by either systemic or combined systemic/mucosal application of vaccines, showing that both immunization strategies induced immune control of virus replication and protected Chinese RM from disease progression with combined systemic/mucosal vaccination [41]. In another pre-clinical study [43], immune-response profiles and protective efficacy of different HIV vaccine modalities, including DNA, protein or both, were evaluated in SHIV89.6P-challenged Chinese RM. All vaccine modalities elicited significant immune responses. Importantly, these responses efficiently suppressed the viral replication that was otherwise sustained at high levels in non-vaccinated Chinese RM [43].

Chinese RM have also been examined for immune-based therapeutic approaches for HIV/AIDS. The current concept of AIDS immune therapy has been focused on cytokine-mediated retrieval of T cell expansion. Studies from different groups examined the impact of recombinant IL-2 (rIL-2) on T cell homeostasis in Chinese RM in the context of SIV infection, and found that administering rIL-2 caused a dose dependent expansion of CD4^+^ and CD8^+^ T cells without affecting viral load, in which both CD4^+^ and CD8^+^ regulatory T cells were upregulated [91]. In addition to IL-2, IL-7, a critical cytokine participating in both thymopoiesis and peripheral T cell homeostasis, has also been tested for its therapeutic effect on HIV infection [92]. An interesting study [93] evaluated the effect of IL-7 as a therapeutic approach on immune reconstitution in SIV-infected Chinese RM. The results showed that IL-7 treatment elevated the number of circulating CD4^+^ and CD8^+^ memory T cells that express proliferation (Ki-67) and activation (HLA-DR, CD25) markers, as well as the naïve T cell pool (CD45RAbright CD62L). This study [92,93] also demonstrated that IL-7 therapy did not counteract the reduced plasma viral load achieved under antiretroviral therapy (ART). However, the prognostic effect of treatments with these cytokines on SIV disease outcome needs further investigation using RM, and could well utilize Chinese RM.

In addition to vaccine- and cytokine-based therapies, other novel therapeutic concepts have also been successfully tested in SIV-infected Chinese RM. It was shown that immune manipulation of delta-gamma T cells by a well-defined HMBPP [(E)-4-Hydroxy-3-methyl-but-2-enyl pyrophosphate)]/IL-2 therapeutic regimen could overcome virus-induced immune suppression and confer immunological benefits during the chronic phase of SIV infection of Chinese RM [42]. The antiretroviral drug, tenofovir disoproxil fumarate (TDF), was effective in blocking SHIV infection of Chinese RM [49]. These observations support the feasibility to utilize Chinese RM as a suitable NHP model for testing novel HIV therapeutic concepts.

## Conclusions

Although the published data have demonstrated that Chinese RM is a suitable macaque host for studies of HIV disease, more extensive investigations on testing of current HIV vaccine approaches in Chinese RM are needed in order to determine if efficacy of these approaches in humans can be predicted in Chinese RM. These future studies should provide further support for the use of Chinese RM for the investigation of HIV disease.

## Abbreviations

AGM: African green monkey; AID50: 50% animal infectious doses; AIDS: Acquired immunodeficiency syndrome; CCL: Chemokine ligand; CCR5: C-C chemokine receptor 5; CD: Cluster of differentiation; Ch RM: Chinese rhesus macaques; CMV: Cytomegalovirus; CTL: Cytotoxic T Lymphocyte; DC: Dendritic cells; HIV: Human immunodeficiency virus; HLA: Human leukocyte antigen; HMBPP: (E)-4-Hydroxy-3-methyl-but-2-enyl pyrophosphate; IFN: Interferon; IL: Interleukin; LN: Lymph node; I.R: Intrarectally; I.V: Intravenously; IVAG: Intravaginally; MHC: Major histocompatibility complex; mtDNA: Mitochondrial DNA; NHP: Nonhuman primate; PMN: Polymorphonuclear neutrophils; SHIV: Simian-human immunodeficiency virus; SIV: Simian immunodeficiency virus; SNP: Specific single nucleotide polymorphisms; TCID50: 50% tissue culture infective dose; TDF: Tenofovir disoproxil fumarate.

## Competing interests

The authors declare that they have no competing interests.

## Authors’ contributions

YZ wrote the manuscript. RB collected information for tables. NH, YP and WZH reviewed and edited the manuscript. All authors read and approved the final manuscript.
